# User engagement in a digital health intervention designed for young people who have experienced technology-assisted sexual abuse (i-Minds trial)

**DOI:** 10.1016/j.invent.2025.100858

**Published:** 2025-07-08

**Authors:** Sandra Bucci, Xiaolong Zhang, Kaja Dabrowska, Amanda Larkin, Ethel Quayle, Matthias Schwannauer, Filippo Varese, Pauline Whelan

**Affiliations:** a Division of Psychology and Mental Health, School of Health Sciences, Faculty of Biological, Medical and Health Sciences, Manchester Academic Health Science, University of Manchester, Manchester, United Kingdom; b Greater Manchester Mental Health NHS Foundation Trust, Manchester, United Kingdom; c School of Health in Social Science, University of Edinburgh, Edinburgh, United Kingdom; d NHS Lothian, Edinburgh, United Kingdom

**Keywords:** Adolescent, Mobile applications, Engagement, Digital health intervention

## Abstract

**Background:**

Technology-assisted sexual abuse (TASA) mostly involves the production and non-consensual sharing of sexual images; however, evidence-based support for young people (YP) who have experienced TASA is scant. Digital Health Interventions (DHIs) have the potential to increase access to support and provide timely therapeutic input in a familiar format to YP. However, studies describing engagement with DHIs is nascent. Our objective is to describe engagement patterns for YP people who used the i-Minds app.

**Methods:**

The i-Minds app is a co-designed mentalisation-based DHI for YP who have experienced TASA. Usage data was collected during the 6-week intervention window using Matomo analytics software and analysed according to the AMUsED framework.

**Results:**

Forty-one participants were onboarded to the app. Of these, 95 % completed the introductory mandatory module, and nearly half completed the remaining three modules. Median duration of app engagement was 33 days. Most participants used the app on weekdays in the afternoon. Demographic variables, namely gender not matching with sex assigned at birth/prefer not to disclose and higher baseline clinical severity were associated with higher app engagement.

**Conclusions:**

Participants showed high module completeness and engagement duration, suggesting the potential for real-world use. Potential participant-level predictors of engagement, such as gender identity and severity of TASA related traumatic stress and emotional distress, were identified. Achieving satisfactory engagement in DHIs is challenging yet necessary for delivering effective interventions. Future studies should explore participant-level predictors of engagement to inform real-world use of DHIs with a diverse sample.

## Highlights

• DHIs have been shown to be safe and acceptable for YP who have experienced TASA.
• In-depth analysis of engagement with DHIs is rarely explored in clinical trials.
• This study found participants in the i-Minds trial engaged well with the app.
• This study demonstrates the real-world implementation potential of the i-Minds app.

## 1 Introduction

Using the internet has become a routine part of daily life for young people (YP), but it can place them at risk for various forms of harm. Technology-assisted sexual abuse (TASA) is one form of online harm that can occur across multiple digitally-enabled platforms and applications. TASA can involve YP being coerced into sharing sexual images of themselves, taking part in sexual activities via webcam or smartphone, or ‘sexting’; these behaviours can bring unique social and psychological harms (Saied-Tessier, 2014; Letourneau et al., 2018). Although multiple factors are likely involved in vulnerability to being exposed to TASA, the ability to accurately estimate others' intentions and motivations (termed mentalising) when engaging in online spaces can be compromised because the usual assumptions based on verbal and non-verbal cues in real life interactions are more opaque when communication takes place online. This in turn can compromise a YP's ability to evaluate risk and accurately mentalise (Gewirtz-Meydan et al., 2019). Digital health interventions (DHIs) have been shown to be a safe and acceptable way to support YP; they are unconstrained by location and time and can facilitate access to intervention strategies when traditional means of support are not available. In addition to understanding how feasible, acceptable, safe, and effective DHIs are with vulnerable YP, it is also important to understand engagement patterns, including how DHIs are accessed, used and the way users interact with a DHI. This can help researchers understand what can be done to improve the user experience and how to make interventions more effective. Engagement with DHIs is associated with therapeutic gains (Gan et al., 2021) but has been noted as a significant challenge in DHIs.

Reporting detailed usage analyses of DHI trials is relatively scant in the digital health literature (Miller et al., 2019), possibly because engagement with an intervention is a complex, multidimensional concept (Yeager and Benight, 2022). Engagement in DHIs is a multifaceted concept that remains one of the field's most persistent and consequential challenges (Lipschitz et al., 2023). It is commonly defined as encompassing uptake, sustained use, and adherence (Lipschitz et al., 2023), though definitions and metrics vary across studies. Yardley et al. (Yardley et al., 2016) emphasise the behavioural, cognitive, and emotional dimensions of engagement, while Perski et al. (Perski et al., 2017) distinguish between the extent of engagement (e.g., frequency or duration of use) and its quality (e.g., depth of interaction or meaningfulness). Despite growing theoretical development, standardised definitions and measurement approaches are still lacking. Behavioural engagement is often operationalised through metrics such as uptake (e.g., downloading and initial use), sustained use (e.g., continued engagement over time), and adherence or completion (e.g., using the intervention as intended in terms of frequency and duration). Importantly, engagement thresholds differ across interventions; there is no single benchmark for what constitutes “completion.” Broader definitions, particularly from the human-computer interaction field, conceptualise engagement as the user's temporal, emotional, and cognitive investment, as well as the ability of the intervention to capture and sustain attention and interest (Torous et al., 2020).

Engagement with DHIs is usually quantified through a blend of objective usage analytics and self-report data. Objective indicators, such as log-ins, clicks, time spent on the platform, and module completion, provide a practical, multidimensional snapshot of user behaviour (Lipschitz et al., 2023). While these metrics can serve as useful proxies for cognitive engagement, they reveal little about users' emotional or experiential responses. To capture those elements, researchers may supplement analytics with self-report instruments that probe affective reactions and whether users act on intervention recommendations. Validated scales such as the User Engagement Scale (O'Brien and Toms, 2010; O'Brien et al., 2018) and the Unified Theory of Acceptance and Use of Technology (UTAUT) Scale (Venkatesh et al., 2003) offer additional approaches for assessing engagement, capturing attention, interest, satisfaction, and subjective adherence. Hassan et al. (Hassan et al., 2023) argue that evaluations must look beyond dropout rates to the quality and continuity of engagement, because sustained interaction is essential for DMHIs to realise their intended benefits. Nevertheless, in routine implementation, backend behavioural metrics remain the most feasible and widely adopted because they are automatically recorded and easily scaled.

i-Minds is a co-designed, theory-driven DHI underpinned by mentalisation principles, designed for YP (12–18 years) who have experienced TASA. Details of the development of the app, our method of co-design, subjective measures of user satisfaction and engagement, and findings from the trial are published elsewhere (Bucci et al., 2023). In summary, we conducted a mixed-methods, non-randomised clinical feasibility study with 46 YP aged 12–18 years exposed to TASA across two UK sites, with a nested qualitative study to understand issues to be addressed in advance of conducting a powered efficacy trial (Bucci et al., 2023). We found that it was possible to recruit and retain participants in a DHI trial of TASA, and that the i-Minds app was safe, acceptable and associated with promising signals of efficacy on key outcomes. User feedback indicated that participants generally had a positive experience using the app, positively increasing their knowledge/understanding of their own mental health and their motivation to address their mental health difficulties. In the current study, we report patterns of engagement among participants who took part in the i-Minds trial over the 6-week intervention window guided by the Analysing and Measuring Usage and Engagement Data (AMUsED) framework (Miller et al., 2019). The framework is used to guide researchers in familiarisation of the intervention, identification of key measures, specification of research questions, and preparation of data for analysis.

### 1.1 Objective

Guided by the AMUsED framework with app usage data collected, we aimed to explore user engagement with the i-Minds app during the i-Minds non-randomised clinical feasibility trial. We specifically aimed to explore: i) content-specific completeness of the i-Minds app; ii) the timing and duration of engagement; and iii) association between baseline characteristics and app engagement.

## 2 Methods

### 2.1 Research design

The methods and results of the i-Minds non-randomised clinical feasibility trial regarding clinical outcomes are reported in the trial protocol (Bucci et al., 2023) and outcome paper (*under review*). This paper focuses on the methods and results relevant to examining engagement patterns during the i-Minds clinical feasibility trial. The pre-registered trial (ISRCTN43130832) was approved by the National Research Ethics Committee (REC) of West Scotland REC 4 (approval number 22/WS/0083) and overseen by an independent oversight committee. All participants gave informed consent.

### 2.2 Participants

Participants were recruited from Child and Adolescent Mental Health Services (CAMHS) in Edinburgh and Greater Manchester, one sexual assault referral centre (SARC) in Greater Manchester, and an NHS-commissioned e-therapy provider. Further details of the recruitment procedure are reported elsewhere (Bucci et al., 2023). Potential participants were eligible to take part in the clinical feasibility trial if they were: i) aged 12–18 years; ii) exposed to TASA and report associated distress; iii) receiving support from CAMHS, SARC or an e-therapy provider and continued to be actively supported by services for the trial duration; iv) willing to use an app about TASA; v) proficient in speaking and writing in English; vi) capacity to consent; and, for e-therapy provider users only, willing to provide their username. YP with insufficient command of English, moderate learning difficulties, or at risk of current or recent suicidality (assessed by referrers) were excluded. Following recruitment, baseline assessments were completed to collect data about participant demographics and clinical characteristics. Participants could use their own smartphone and download the i-Minds app with support from a researcher (if requested) or they could opt to borrow a smartphone with the app preloaded. We asked participants to return the loaned handset at study end. Data network charges were paid for the intervention period, as was completion of research assessments.

### 2.3 Intervention

The i-Minds app is a modular DHI available over 6-weeks. The app is designed to support help-seeking YP-OSA (i.e. those already under the care of services or an e-therapy provider) better mentalise in the online environment and is intended to be used as a stand-alone app. App content is organized into four modules: 1) mentalisation (mandatory module to complete before access to other modules is permitted); 2) psychoeducation (technology, relationships, sexual experiences); 3) emotional and mental health; and 4) trauma (focusing on the psychological and emotional consequences of trauma, including recognising and responding to ‘triggers’, addressing and improving feelings of guilt and shame, and reducing maladaptive avoidance). In addition to module content, participants can access a repository of multi-media material designed to support learning and promote engagement, including videos and audio exercises, a diary function, links to podcasts, blog posts and other useful links, real-life stories of recovery, emergency and safeguarding contacts, relaxing/breathing/mindfulness exercises, soothing visual images, and inspiring quotes. Topics areas are visually represented and navigation through the app uses a tree motif; the tree grows leaves as segments and modules are completed. Users are sent a daily prompt in the form of a standard app notification (at 5 pm), designed to prompt engagement, with a further notification three hours later (at 8 pm) if the initial prompt did not result in the use of the app; users can also self-initiate use at any time. Screenshots of the i-Minds app modules and their content are shown in Supplementary Fig. 1 and Supplementary Table 2.

### 2.4 Analysis

The AMUsED framework was used to systematically guide analysis of usage data (Miller et al., 2019). The AMUsED checklists were completed prior to analysis (see Supplementary Table 1). Stage 1 AMUsED checklist was used to specify specific workflow and content of the intervention, review data accrued, and understand the underlying mechanism of the intervention. Stage 2 AMUsED checklist was used to review usage metrics and available baseline characteristics, how they could be associated and to develop the research questions. Stage 3 AMUsED checklist was used to understand the data format, specify and plan tasks in order to prepare the data for analysis, and review available tools for analysis.

App usage data was collected using Matomo analytics, an open analytics platform (Matomo, 2023). Matomo analytics collects a large variety of usage indicators, including user-specific measures such as time spent using the app (daily, overall), and number of visits and actions (daily, overall), as well as aggregate measures, such as total time spent using the app by all users during each day of the week or each time of day.

Descriptive statistics were used to characterize engagement frequency, duration and type of participants. The associations between baseline demographic and clinical characteristics and app engagement were assessed using Spearman's correlation (continuous variables), independent *t*-tests (binary variables), or ANOVA (categorical variables). Due to small sample sizes in certain demographic categories, variables were regrouped to enable meaningful comparisons (i.e. gender matches sex assigned at birth, level of education, employment status, relationship status), while those that could not be regrouped or still had a small sample size after regrouping were not analysed (i.e. ethnicity, living status). Analyses were performed in R (version 4.3.2) (R Core Team, 2021). The *P* value threshold was set to 0.05.

## 3 Results

### 3.1 Sample characteristics

Of the 147 young people screened for eligibility, 72 referrals were made to the trial and 65 participants were deemed eligible; 46 young people consented to take part in the i-Minds clinical feasibility trial. Three participants withdrew consent prior to completing baseline assessment, leaving 43 participants completing baseline assessment and 41 participants onboarded to the app. Demographic and clinical characteristics of the 41 young people onboarded to the i-Minds app are reported in Table 1 (demographics of the wider i-Minds sample are reported elsewhere; *Bucci* et al.*, under review*). The average age of participants was 15.2 (SD = 1.45) years. Most identified as female (70.73 %) and White British (97.56 %). A notable percentage of participants (14.64 %) identified as non-binary/third gender or preferred not to disclose their gender identity, and 21.95 % reported their gender did not match their sex assigned at birth or preferred not to disclose their gender. Most participants were living with parents (95.12 %), currently a student (85.37 %), single (78.05 %) and did not use apps to help with their mental health (87.8 %). Two-fifths (39.02 %) of participants were taking medication, and 60.98 % were receiving psychological support, with just over half having received a psychiatric diagnosis. As per eligibility criteria, all participants reported a history of distress associated with TASA. Most participants accessed the i-Minds app via their own phone; 4.88 % (*n* = 2) of the sample requested a study handset.

**Table 1:** Baseline characteristics and service use (*N* = 41).

Characteristic		N (%)
Age (years)	Mean (min-max)	15.2 (12–18)
Age group	12–14	12 (29.27 %)
	15–18	29 (70.73 %)
Gender	Man / Male (n/%)	6 (14.63 %)
	Woman / Female (n/%)	29 (70.73 %)
	Non-binary / Third gender	3 (7.32 %)
	I would rather not say / unsure	3 (7.32 %)
Gender matches sex assigned at birth	Yes	32 (78.05 %)
	No	8 (19.51 %)
	Prefer not to say	1 (2.44 %)
Ethnicity	White British	40 (97.56 %)
	Asian/Asian British	1 (2.44 %)
Level of education	Primary school	28 (68.29 %)
	Secondary school	12 (29.27 %)
	Further education	1 (2.44 %)
Living status	Living with parents	39 (95.12 %)
	Living alone	1 (2.44 %)
	Other	1 (2.44 %)
Career status	Yes	6 (14.63 %)
	No	35 (85.37 %)
Employment status	Student	35 (85.37 %)
	Part time / casual job	3 (7.32 %)
	Voluntary job	3 (7.32 %)
	Other	1 (2.44 %)
Relationship status	Single	32 (78.05 %)
	Partnered	8 (19.51 %)
	Rather not say	1 (2.44 %)
Study phone	Own phone	39 (95.12 %)
	Borrowed phone	2 (4.88 %)
Deprivation Index	Range	Scotland: 248–6933 England: 4282–32,503
Baseline service use and use of internet / being online		N (%)
Currently supported by CAMHS	No	2 (4.88 %)
	CAMHS / Child & Adolescent Mental Health Team	32 (78.05 %)
	Sexual Assault Referral Centre (SARC)	2 (4.88 %)
	e-therapy provider	2 (4.88 %)
Use apps to support mental health	Yes	5 (12.2 %)
	No	36 (87.8 %)
Have been in psychiatric hospital / ward	Yes	3 (7.32 %)
	No	38 (92.68 %)
Take medications	Yes	16 (39.02 %)
	No	25 (60.98 %)
Receive psychological support	Yes	25 (60.98 %)
	No	16 (39.02 %)
Have received a diagnosis	Yes	22 (53.66 %)
	No	19 (46.34 %)

### 3.2 Engagement with i-minds features

**Fig. 1:**
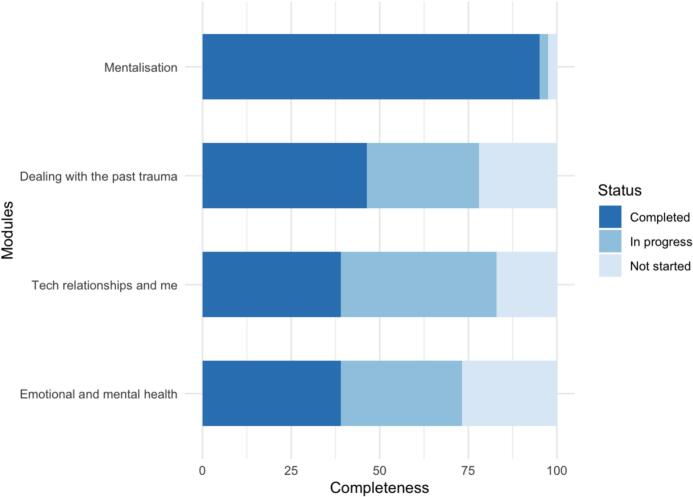
The completeness of the i-Minds modules.

Of the 41 YP onboarded to the app, 95 % users (39/41) completed the mandatory mentalisation module. For the three remaining modules, the trauma module had the most completions (46 % (19/41) users completed the module), followed by the psychoeducation and emotional and mental health modules (39 % (16/41) users completed each module). Additionally, 32 %, 44 %, and 34 % of users accessed (but did not complete) the trauma, psychoeducation, and emotional and mental health modules, respectively. A breakdown of completion status of each module is shown in Fig. 1. The total time spent on each module, multi-media resources, and diary is displayed in Supplementary Fig. 2. Of the four intervention modules, the median total time spent on the mandatory mentalisation module was the lowest (2.48 min, IQR 1.03–4.87, range 0.05–22.15), whereas the median total time spent on the trauma module was the highest (7.87 min, IQR 3.98–12.66, range 0.08–56.22). Besides the intervention modules, the median total time spent on the multi-media resources and diary was 4.67 min (IQR 2.27–13.95, range 0.02–27.32) and 1.68 min (IQR 0.81–4.22, range 0–15.52), respectively.

### 3.3 Duration and timing of engagement

**Fig. 2:**
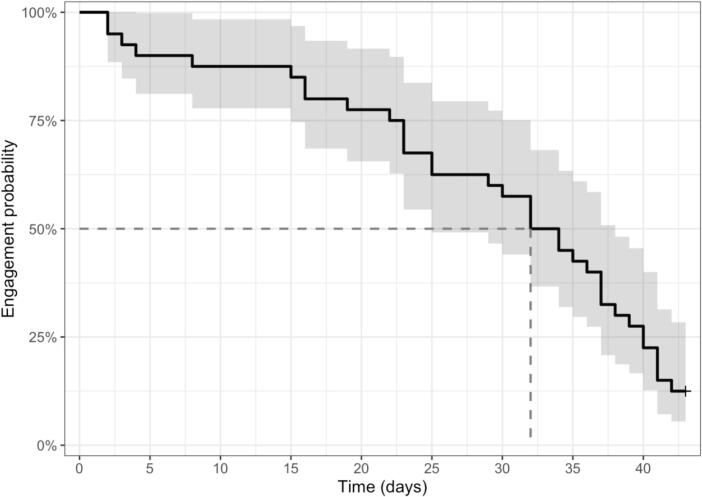
Kaplan-Meier plot of engagement over six weeks.

Fig. 2 shows the probability of engagement with the app across six weeks. The median survival time was 33 days (95 % CI 25–38). The distribution of daily total time spent on the app in each week is shown in Supplementary Fig. 3. The median total time spent on the app ranged from 1.1 min (IQR 0.27–5.43, range 0–34.15) in week 3 to 5.78 min (IQR 1.22–14.53, range 0–88.52) in week 1. Most participants spent relatively less time on the app compared to the median time spent, whilst a small number of participants spent notably longer using the app compared to their peers.

**Fig. 3:**
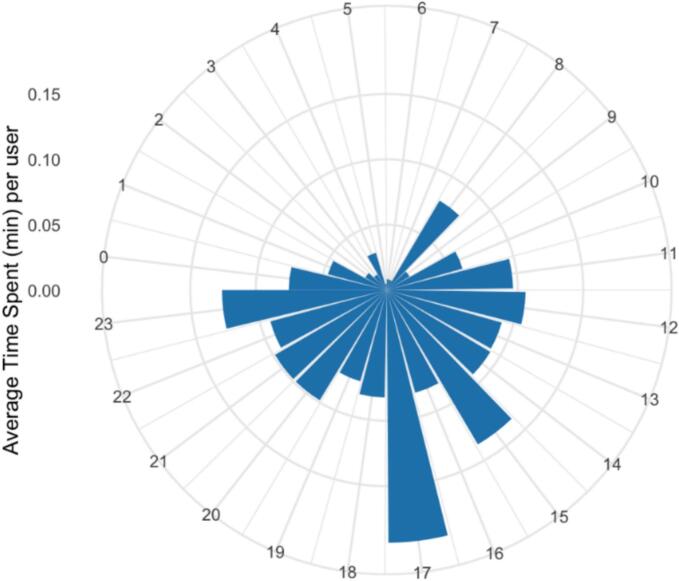
Average time spent using the app in a day.

**Fig. 4:**
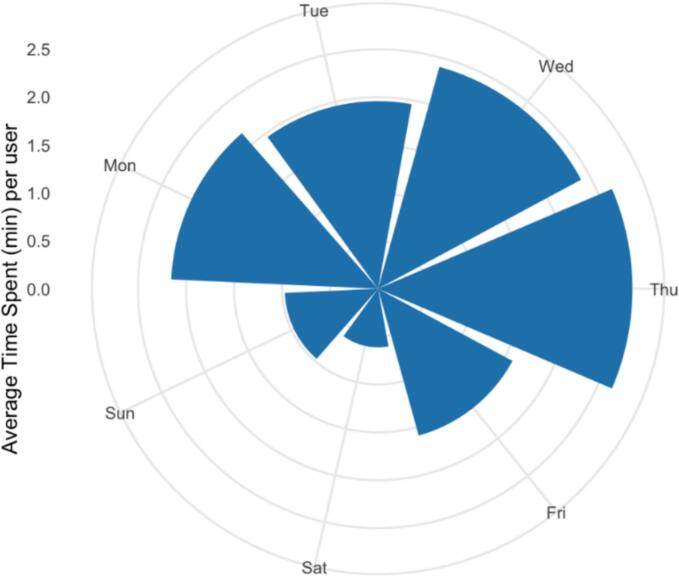
Average time spent using the app in a week.

Fig. 3 shows the average time spent per user in a day. Participants mostly interacted with the app in the afternoon; 5 pm was the highest use period in line with the app prompt schedule. As shown in Fig. 4, participants spent more time on the app on weekdays than weekends; more specifically, participants spent most of time on the app on Thursday, followed by Wednesday and Monday.

### 3.4 Factors associated with engagement

We conducted correlation analysis of baseline demographic and clinical characteristics and engagement data. Among the demographic factors, participants who identified their gender as not matching with the sex assigned at birth or preferred not to disclose spent more time on the app (*t* = −4.83, *p* < 0.001); no significant associations were found between other demographic features and app engagement: age (rho = 0.07, *p* = 0.664), gender (F = 2.36, *p* = 0.087), level of education (*t* = −0.11, *p* = 0.91), career status (*t* = 0.85, *p* = 0.401), employment status (*t* = 0.01, *p* = 0.993), and relationship status (*t* = −0.21, *p* = 0.834). Of the baseline clinical assessments, longer total time spent on the app was associated with a higher baseline intrusion score (rho = 0.33, *p* < 0.05) on the Child Revised Impact of Events Scale (CRIES) (Perrin et al., 2005), and anxiety (rho = 0.36, *p* = 0.025), depression (rho = 0.39, *p* = 0.015) and total emotional distress scores (rho = 0.36, *p* = 0.026) of the Revised Child Anxiety and Depression Scale–25 item version (RCADS–25) (Ebesutani et al., 2012).

## 4 Discussion

### 4.1 Principal findings

This study aimed to understand usage patterns of the i-Minds app designed for YP who have experienced distress associated with TASA in a clinical feasibility trial. We found that, of the four intervention modules, 95 % participants onboarded to the app completed the mandatory mentalisation module, and the remaining three modules were completed by nearly half of the participants. Survival analysis of app disengagement indicated that the median survival time was 33 days. Most participants used the app in the afternoon and spent more time on the app on weekdays than weekends. Correlation analysis showed that participants who reported their gender identity differing from their sex assigned at birth/preferred not to disclose, higher levels of intrusion and emotional distress of TASA at baseline spent more time using the app.

Engagement of DHIs in real-world settings remains limited, and the reduction of usage over time was commonly reported (Linardon and Fuller-Tyszkiewicz, 2020) (Torous et al., 2021). As the nature of intervention and definition of engagement varies across studies, a direct comparison of engagement is difficult. A recent systematic review on engagement of smartphone-delivered interventions found that the mean percentage of participants completing the designed intervention content was between 34 % and 64 % for a range of mental health conditions, including depression, anxiety, stress, and general mental health (Linardon and Fuller-Tyszkiewicz, 2020). Another systematic review on YP's engagement with DHIs in general showed that the completion rates of intervention content varied hugely across studies, ranging from 3.6 % to 100 % (Whitehead et al., 2024). Considering we did not incentivise participants to engage with the app, i-Minds demonstrated relatively high engagement compared to other DHIs by successfully achieving 95 % of participants completing the mandatory module and nearly half of the participants completing the remaining three modules. Moreover, the trajectory of engagement with i-Minds declined steadily, whereas some DHIs trials showed a drastic engagement reduction over time (Flett et al., 2019) (Morrison et al., 2017). Several design features may explain this favourable pattern. First, mandating the Mentalisation module ensured exposure to core therapeutic principles but may also have contributed to lower uptake of subsequent optional content. Second, a carefully calibrated daily push-notification schedule appeared effective, consistent with evidence that timely, well-designed reminders can sustain use without inducing alert fatigue (Bidargaddi et al., 2020). Finally, the co-designed graphical user interface, featuring a youth-friendly colour palette and visual displays (a tree motif; the tree grows leaves as segments and modules are completed; video material), and minimising text where possible, was intended to maximise usability and aesthetic appeal, both recognised factors influencing engagement in DHIs (Bidargaddi et al., 2020) (Perski et al., 2017). Taken together, these elements provide important context for interpreting the i-Minds engagement outcomes and offer practical guidance for future intervention design.

Identifying an approach to record and report on usage/engagement data for DHIs can be complex, as engagement is a multidimensional concept that includes behavioural, cognitive, and emotional components (Yeager and Benight, 2018). However, many studies of usage/engagement of DHIs do not report these in a systematic manner, despite frameworks proposed by researchers to investigate engagement and its impact (Yeager et al., 2018) (Short et al., 2015) (Perski et al., 2017). This may be due to the lack of implementing appropriate tools to facilitate the systematic identification, extraction, and analysis of usage data (Miller et al., 2019). To address this issue, we employed the AMUsED framework to support a transparent approach to analysis and reporting. The AMUsED framework contains comprehensive checklists establishing a systematic guide for analysing usage data by drawing together potential measures of usage and identifying which are meaningful to the intervention, generating specific research questions to act as testable hypotheses, and supporting data preparation and selection of methods for analysis (Miller et al., 2019). Additionally, we used Matomo to collect objective data to support the evaluation of app usage; using a contemporary analytics software tool enabled us to collect a huge volume of detailed usage and engagement user data, which is one of the significant advantages of such tools.

Understanding the relationship between engagement and mental health outcomes is a critical step for optimising DHIs to be used more effectively. A recent meta-analysis showed that more frequent engagement with DHIs was associated with better mental health outcomes (Gan et al., 2021). However, due to the non-randomised study design, we were not able to determine the ‘effective dose’ of the i-Minds app (i.e. how much ‘engagement’ with the app is sufficient to bring about clinical change). Instead, we found app engagement was significantly associated with participants' gender identity and the level of distress caused by TASA at baseline. This might support the idea that, when app content is relevant to the user, engagement increases (Borghouts et al., 2021) (Garrido et al., 2019). A future RCT is warranted to further explore the relationships between engagement with i-Minds and its potential clinical benefits.

We did not use the usage and engagement analytics in real-time to support engagement (e.g. to identify when a participant had stopped engaging with the app and to encourage them to re-engage). However, in future, the app could provide a nudge to the YP if engagement drops below pre-defined/agreed threshold or engagement analytics could be used to invoke human support (e.g. call from supporting therapist).Our analysis of usage/engagement data can help us understand how digital tools may reach groups that may have traditionally been disadvantaged from accessing mental healthcare and as such may therefore have potential to address and reconfigure existing health inequalities.

### 4.2 Strengths and limitations

This study explored objective and detailed usage data from the i-Minds app to characterize engagement over time. We did not define a priori hypotheses or pre-specified thresholds for engagement; the study was exploratory in nature. As the sample was largely White British help-seeking females, it is not clear how transferable findings around engagement with a DHI of this nature are to other groups of YP who have experienced TASA. Clinicians made decisions about YP they would refer to the i-Minds trial. Decisions were based on factors such as whether a young person was well and ready enough to take part, or whether the DHI ‘fit’ with the treatment model they were using. It is possible that the trial attracted more digitally literate YP with more positive attitudes towards DHIs than the broader population of YP who have experienced TASA. Furthermore, usage analysis is based on automatically collected analytics from the app. Engagement is a complex concept that ideally requires integrating adjacent constructs, including predictors, behavioural outcomes and other measures, such as attention, affect and interest. Nonetheless, usage data analysis is still valuable to explore how the intervention was utilised, which was the aim of this study. However, the results should be interpreted with caution as inactive usage may exist and lead to an overestimation of engagement. While certain aspects of the i-Minds trial were designed to reflect real-world conditions, such as lack of financial incentives for app usage, others, such as paid network charges for the intervention window and provision of phones and data plans, may limit generalisability. Finally, the measures administered in the i-Minds trial, though not included in analyses reported here, might have influenced engagement with the app.

### 4.3 Conclusions

There are currently no evidence-based assessments or interventions for YP exposed to TASA. Achieving satisfactory engagement in DHIs is challenging yet necessary for delivering effective interventions. This study analysed usage data from the co-designed, mentalisation-based DHI with YP who have experienced TASA to provide a detailed analysis of patterns of engagement and attrition. We have demonstrated that a sizeable proportion of participants used the app for the duration of the study, suggesting the potential for real-world implementation of the i-Minds app. Gender identity differing from sex assigned at birth, higher baseline intrusion and emotional distress related to TASA were identified as factors associated with higher engagement levels. Future studies should further explore the relationships between baseline demographic and clinical characteristics and DHIs engagement and take advantage of opportunities to exploit detailed usage data to inform the real-world implementation of DHIs with diverse samples in practice.

## CRediT authorship contribution statement

SB, XZ, and KD drafted the manuscript. XZ conducted the data analysis. SB, FV, MS, and EQ conceptualised the study, secured funding, and led the development of the i-Minds app. MM is responsible for engineering the i-Minds app. PW led the participatory design work. SB, EQ, MS, FV and AL supervised data collection. All authors critically revised the manuscript for intellectual content and approved the final version of this protocol.

## Declaration of competing interest

Unrelated to this project, SB is co-founder of CareLoop Health Ltd., and PW is Chief Operating Officer and co-founder of CareLoop Health Ltd., which develops and markets digital therapeutics for schizophrenia and a digital screening app for postnatal depression. SB also reports research funding from the National Institute for Health and Care Research, Wellcome Trust, and Medical Research Council. FV reports research funding from the National Institute for Health and Care Research.

## Data Availability

The data set generated in this study is not publicly available owing to ethical restrictions for personal health information; however, limited, deidentified data may be made available from the corresponding author on reasonable request.

## Appendix A Supplementary data

Supplementary material Image 1
