# Supplementary material for: User engagement in a digital health intervention designed for young people who have experienced technology-assisted sexual abuse (i-Minds trial)
Source: Internet Interv. 2025 Jul 8;41:100858. doi: 10.1016/j.invent.2025.100858 (PMC12274295; doi:10.1016/j.invent.2025.100858)
Supplement: Supplementary file 1 — Supplementary material [file mmc1.docx]

**Supplementary Table 1.** AMUsED checklists.

*Stage 1 Checklist.*

| Familiarisation with the data – identifying variables | |
| --- | --- |
| Generic questions by data type | Intervention Name: |
| **1. Intervention characteristics. Data for intervention architecture and content.** | |
| **1.1. Workflow. Intervention structure and expected participant interaction and navigation through the intervention.** | |
| How many logins/sessions are available? | There is no limitation on the number of available sessions. |
| When are they available? | During the whole study period. |
| Are new sessions released depending on time elapsed or task-completion? | Once the mandatory module is completed, the remaining content is made available to the user. |
| Are there limitations on the availability of the intervention? | The study duration was 6 weeks, but the intervention can be accessed anytime during this time window. |
| Is the purpose of a session to collect self-report measures and/or use the intervention? | The purpose of the session is to use the intervention. |
| When is the intervention considered to be finished? | The intervention was designed to be used as a support tool as needed, without a specific completion task. The intervention can be accessed by the user as many times as needed. |
| What prompts are used to encourage usage (e.g. emails, texts, notifications) and when are they sent? | Participants received a daily prompt in a form of a notification to encourage app use. If a participant could not interact with the app at the time of the prompt, they received a reminder notification 3 hours later. This daily prompt was not sent if the participant had already used the app on that day. |
| Does the intervention contain ‘tunneled’ (compulsory) sequences of pages which users have to view to move forward? | The mandatory module is a tunnelled sequence of pages. Once the mandatory module is completed, the remaining content is made available to the user, which can be viewed in any order. |
| Are users able to select linked components they wish to view, and avoid others? | Excluding the mandatory module, users are free to interact only with components they wish to view. |
| **1.2. Content. Content available within the pages of the intervention.** | |
| What are the components available? | Educational modules: “Mentalisation”; “Tech, relationships and me”; “Emotional & mental health”; “Trauma”. Self-reported: “Diary”. Further resources: “Resources”. |
| What is the aim of each component and are they based on underlying theoretical constructs? | Intervention is based on the mentalisation theory, which is the topic of the first mandatory module. The rest of the modules aim to support the user with the management of the consequences of their experiences, such as anxiety and depression. |
| In what order is it anticipated the components will be used? | Once the mandatory module is completed, the user is free to access the rest of the components as needed. |
| What interactive features are available (e.g. forums, videos, printable information)? How long should they take to complete? | The intervention contains: videos, text, quizzes, a diary, useful links. Each video, text and quiz should take no more than few minutes to complete. |
| Are all components/features available to all users throughout the intervention or are some tailored for specific times or users? | All components are available to all users at all times, assuming they have completed the mandatory module. |
| Which pages are for collecting self-report measures or for administrative purposes (e.g. questionnaires, login, password change)? | The “Diary” page collects self-report measures, although it was designed solely for users benefit and is not used in the study. |
| Are there specific pages to mark the start and end of sessions? | No. |
| Which pages contain BCTs (e.g. information, planning, feedback) and what are they? | All previously mentioned educational modules contain BCTs in form of videos and text, some also include quizzes. Additional resources available from “Resources” page contain text and links. |
| In which sessions are they available? | They are available in all sessions. |
| Can specific BCTs be identified on particular pages or groups of pages? How many groups are there? | No. |
| Do any of the pages have response options to collect information in addition to baseline/follow-up measures? What data is collected? | “Diary” page collects self-report measures, although it was designed solely for users benefit and is not used in the study. |
| **2. Accrued data. Data collected during an intervention.** | |
| **2.1. Self-report. Users’ self-reported responses collected across various stages of the trial.** | |
| When are self-report questionnaires collected (e.g. weekly logins, monthly symptom information, follow-up at 6 months)? | Follow up. |
| What demographic information is available (e.g. age, gender, education)? | Date of birth, gender, deprivation index calculated by postcode, ethnicity, education level, carer status, employment status, relationship status, mental health support received, previous use of apps for mental health, medications prescribed for mental health, and any mental health diagnoses received. |
| Which measures are specifically related to the target behavior and how often are they collected? | Mentalisation ability scores (RFQ Certainty, RFQ Uncertainty). Collected at baseline and follow-up. |
| Which measures of beliefs influential on the target behavior are collected and when? | Mentalisation ability scores (RFQ Certainty, RFQ Uncertainty). Collected at baseline and follow-up. |
| Are measures of health collected (e.g. conditions which may impact on target behavior or are co-morbid) and psychosocial factors (e.g. anxiety, illness perception, motivation)? | RFQ-Y, RCADS-25, CRIES, DERS-SF, ISM. CD-RISC-10, collected at baseline and follow-up. PRIUS, ECR-RC, collected at baseline. |
| Are additional measures collected at follow-up (e.g. satisfaction, adherence)? | Engagement, Functionality, Aesthetics, and Information collected using the uMARS, as well as questions about participants’ star rating of the app, and whether they would recommend it and how often they would share information from the app. There was also a scale that assesses the perceived impact on their mental health. |
| **2.2. Log-data. Information automatically collected through engagement with an intervention.** | |
| What data is the software platform able to record? | Time spent using the app, number of sessions, completeness of modules, activity log of each user. |
| Are number, date and time of logins available by individual user? | Yes. |
| Are individuals’ total durations of usage accessible? | Yes. |
| Are the number and time of usage prompts recorded? | No. |
| Are there details for which pages were viewed, the sequential order and time spent viewing? | Yes. |
| **2.3. External data. Data collected independently but alongside intervention usage.** | |
| How and where is the data collected (e.g. GP or support staff notes, lab reports, other digital data such as activity or location trackers)? | N/A – no external data collected. |
| What data is collected? | N/A – no external data collected. |
| Which of these measures relate to or may impact on the target behavior? | N/A – no external data collected. |
| **3. Contextual data. Data indirectly related to the running of the intervention which may be influential over usage and analysis.** | |
| **3.1. External factors. Structures and events which may influence participation in the intervention.** | |
| How are users recruited to the intervention? | Participants were identified by clinicians through child and adolescent mental health teams, a sexual assault referral centre and an e-therapy provider. They were given information about the project and asked if they would be interested in taking part. |
| Did any specific large-scale events, with the potential to impact on the intervention, occur during the period of the intervention (e.g. changes in treatment, health campaigns, illness outbreak, technical issues with the intervention)? | Minor technical issues and a case of loss of access due to external factors. |
| **3.2. Previous theory and findings. Results of behavioral analyses carried out during intervention development (e.g. logic models), and analyses of clinical outcomes if available.** | |
| What are the hypothesized mechanisms of the intervention (e.g. as specified in the intervention’s logic model)? | The intervention targets mentalising ability to reduce risk of future harm. The intervention also supports the management of the consequences of participants experiences, such as anxiety and depression. |
| Which factors are identified as important in qualitative research, and can they be related to the variables collected in the trial (e.g. preferences for specific pages)? | Clinical severity of mental health issues, mentalisation ability scores (RFQ Certainty, RFQ Uncertainty), age. All identified as important in accessing overall usage. |
| Which variables are identified as relating to outcomes (e.g. behavioral determinants, theoretical constructs, health factors)? | No behavioural outcome data available. |

*Stage 2 Checklist.*

| Selecting usage variables and generating research questions | |
| --- | --- |
| Generic questions | Intervention Name: |
| **1. Descriptions of usage variables. Which usage variables are relevant to the intervention and in which format (e.g. number of users/sessions, duration, percentage of total, dichotomous)?** | |
| Completing intervention/trial period (stage1; 1.1 & 2.2). E.g. How many users complete the trial? What is the average time taken to complete? | How many users completed the mandatory module? How many users accessed all the educational modules? |
| Logins or sessions where the intervention was accessed (stage 1; 1.1 & 2.2). E.g. How many users start/complete each login/session? How long does it take to complete each session? How many pages are viewed within the session? Which session has the highest proportion of pages viewed, or duration of time spent on it? | N/A |
| Date of login and usage. E.g. When do users login? What time of year? Are there changes in frequency of logins? | What day of the week is the intervention accessed most frequently? Is there a difference in usage during weekdays vs weekend? |
| Time of day of login and usage. E.g. What time of day is usage? Are users more likely to spend longer on the intervention at certain times? | What time of day is the intervention accessed most frequently? |
| Days/weeks of usage (stage1; 1.1 & 2.2). E.g. For how many days/weeks out of the total is the intervention accessed for? How many times within a week is the intervention accessed? Are there repeated uses within the same day? | How many days out of the 6 weeks duration of the study is the intervention accessed for? How many times a day/a week is it accessed? |
| Response to prompts/notifications (e.g. requests to login, email, text, upload data) (stage1; 1.1 & 2.2). E.g. How many responses are sent? How long after receiving notification do users take to log-in or respond? | N/A. |
| Features/linked menu components used (stage 1; 1.1, 1.2 & 2.2). E.g. How many features/components are accessed? How many users access each one? Which are completed and by how many users? Which feature/component has the highest proportion of pages viewed or time spent? What order are they viewed in? Is this the anticipated order? Which have the highest proportion of drop-out? | How many users completed each accessed/completed each component? How many users accessed each resource? How many users accessed the diary/how many entries? Which component has the highest amount of drop out? |
| Revisiting components/features (stage 1; 1.1, 1.2 & 2.2). E.g. Are any used repeatedly? How many times are they revisited, and for how long? Which are most revisited? | Which is the most visited module? How many times was each module accessed in total? |
| Type of content/BCTs used (excluding administration pages) (stage 1; 1.2 & 2.2). E.g. How many groups of pages with similar content are accessed and by how many users? How many pages within the group are used? How many users view each page? Which groups of pages have the highest proportion of views? Which pages are viewed at each login, and when is the largest amount of pages viewed? Which pages have higher drop-out? | How many pages within each module are viewed on average? |
| Completing ongoing measures (e.g. monthly questionnaires, response options within content pages, uploading information or text responses) (stage 1; 2.1 & 2.2). E.g. How many users complete ongoing measures? When do they complete them? Do they also access the intervention at that time? | N/A |
| External device usage (e.g. wearables and other sensor technologies) (stage 1; 2.3). E.g. How much time is spent with the device? How many times is it used? What number of days/weeks is it used for? | N/A |
| **2. Relationships between usage and participant characteristics. Are users’ demographic, physical or psychosocial characteristics at baseline related to intervention usage?** | |
| Are any characteristics at baseline related to usage? E.g. Is anxiety associated with revisiting features? Is current health related to usage of external devices? Are users who spend more time on the intervention older than those who spend less time? Which characteristics are associated with drop- out? | Is clinical severity related to usage of modules specifically aimed at supporting the management of related mental health issues?  Is age related to the overall time spent using the intervention? |
| Are any contextual factors associated with usage (stage1; 3)? E.g. Is manner of recruitment related to usage? | Not for investigation. |
| Do high/low users differ by other usage factors? E.g. Do users who spend more time on the intervention view more types of content than users who spend less time? Is usage of an external device related to intervention usage? | Not for investigation. |
| **3. Relationships between usage, behavioral determinants, and target behaviors. Which usage variables are associated with follow-up measures for target behavior and behavioral determinants? Which usage variables help explain changes in behavior across the intervention?** | |
| Are baseline measures for behavioral determinants/target behavior related to usage? E.g. Is the number of days the intervention is used for related to a behavioral determinant? Do users with low target behavior spend less time on the intervention? | Is the time spent using the app related to age?  Is the time spent using the app related to clinical severity scores? |
| Which usage variables are related to behavioral determinants/target behaviors and at follow-up? E.g. Do users who view a group of pages containing a specific BCT score higher/lower for the associated behavioral determinant? Is completing/not completing a particular component associated with target behavior at follow-up? Is the time spent on a session related to target behavior? | Outcome measures not available. |
| Is usage associated with measures for acceptability/satisfaction at follow-up? E.g. Are high levels of satisfaction associated with accessing more pages? Do users with low satisfaction spend less time using external devices? | Acceptability/satisfaction not for investigation. |
| Do users who report positive changes in behavioral determinants/target behavior from baseline to follow-up use the intervention differently to those who do not? E.g. Do users who report positive increases in a behavioral determinant view more pages from a specific component containing an associated BCT? Do users who report positive behavior change spend more time on the intervention? | Outcome measures not available. |
| Are relationships between usage and target behavior moderated by demographic, psychosocial or health factors? E.g. Is the relationship between time spent on the intervention and target behavior altered when moderated by anxiety? | Outcome measures not available. |
| What level of usage is necessary for ‘effective engagement’? E.g. Do outcome measures plateau after viewing certain content, or after a certain amount of time or sessions completed? | Outcome measures not available. |

*Stage 3 Checklist.*

| Preparation for analysis | |
| --- | --- |
| Generic questions | Intervention Name: |
| **1. Resources** | |
| What is the timeframe for completing the analyses? | 6 weeks. |
| What resources are needed? E.g. additional research time, expertise | Clinical expertise (i-Minds team). |
| Is a plan of analysis already available? How does the analysis plan developed using the framework compare to that plan? Are changes or updates needed? | No plan of analysis regarding app usage available. |
| Is ethical clearance in place to carry out usage analyses? | Yes. |
| **2. Selecting types of analysis and analytical software** | |
| Will the usage data be triangulated with qualitative data? | No. |
| What analytical tools are available? | R studio. |
| Is there sufficient statistical power to answer the planned research questions? | Analysis mostly consists of descriptive analytics. Not enough statistical power to analyse correlation/draw inference. |
| Can the selected measures of usage be analyzed using the available tools? Is bespoke software necessary (e.g. visualisation techniques)? | Selected measures can be analysed using the available tool. |
| **3. Data preparation** | |
| When is the data available? | At the time of analysis. |
| Is the data raw or has it been used/cleaned previously? | Data raw. |
| How many datasheets are there? Will these need to be amalgamated? | Multiple data sheets, need to be merged for a) user-specific analysis b) aggravated measures analysis. |
| Is the data structured to work with the tools available? What formats are the datasheets in (e.g. excel, .csv) and will they need converting for analysis? | Data available in .csv, .xlsx and .xml. .xml formats need converting. |
| What preparation does the data need (e.g. cleaning, anonymizing)? | Data already anonymised. Multiple data sheets need to be merged for a) user-specific analysis b) aggravated measures analysis. |
| Are all variables readily available or will they need extracting/transforming/recoding? | All variables readily available. |
| Is the data in the right format to answer the research questions? Will it need adapting (e.g. continuous variables changed to categorical)? | Date of birth needs adapting to age. All other data in the right format. |

**Supplementary Figure 1.** Screenshots of the i-Minds app.


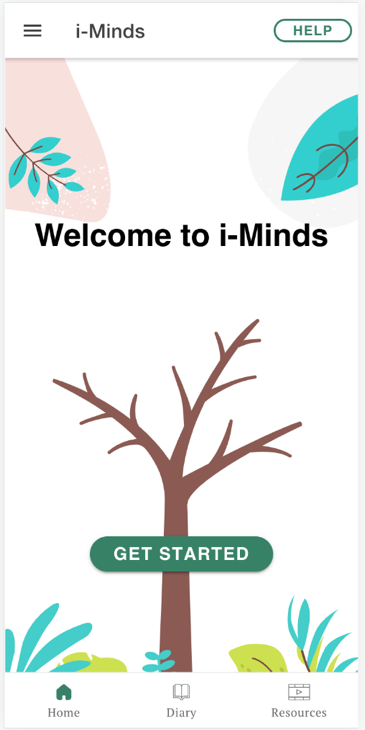

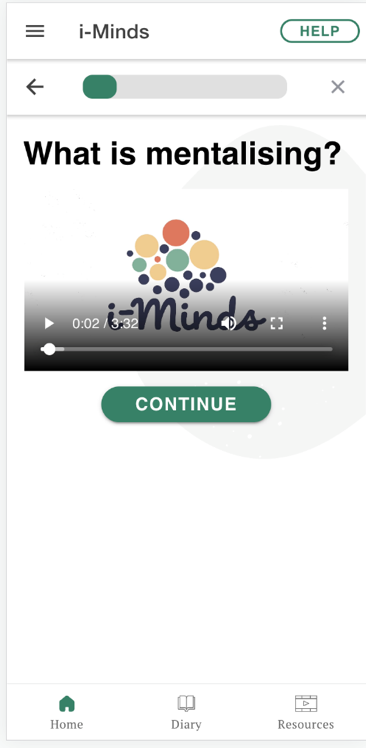

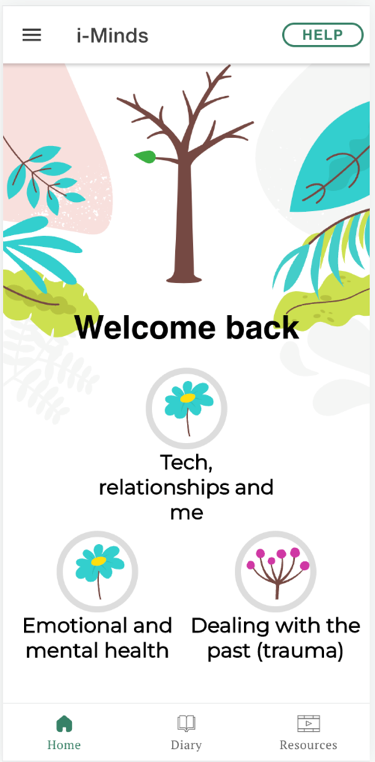


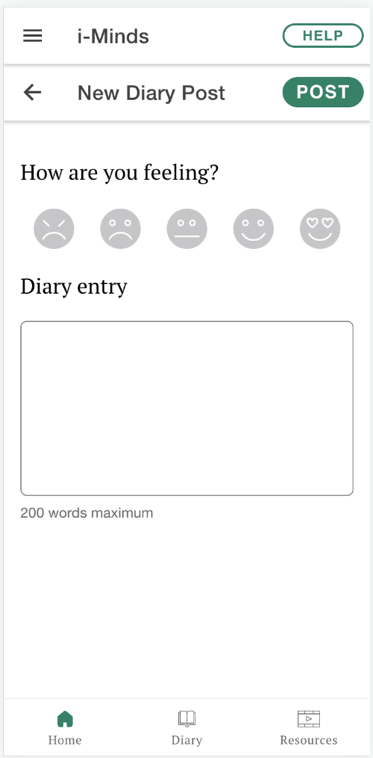

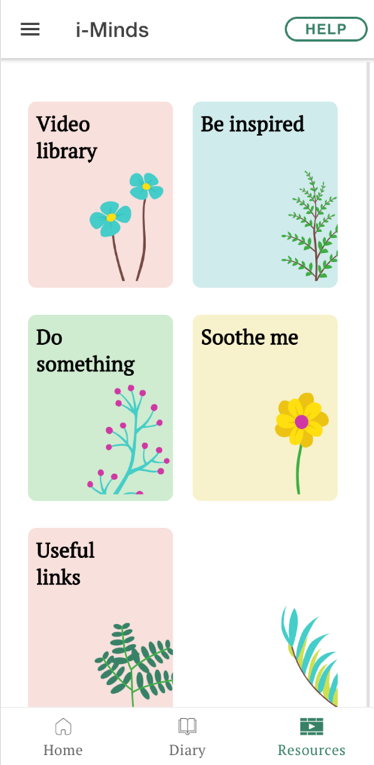

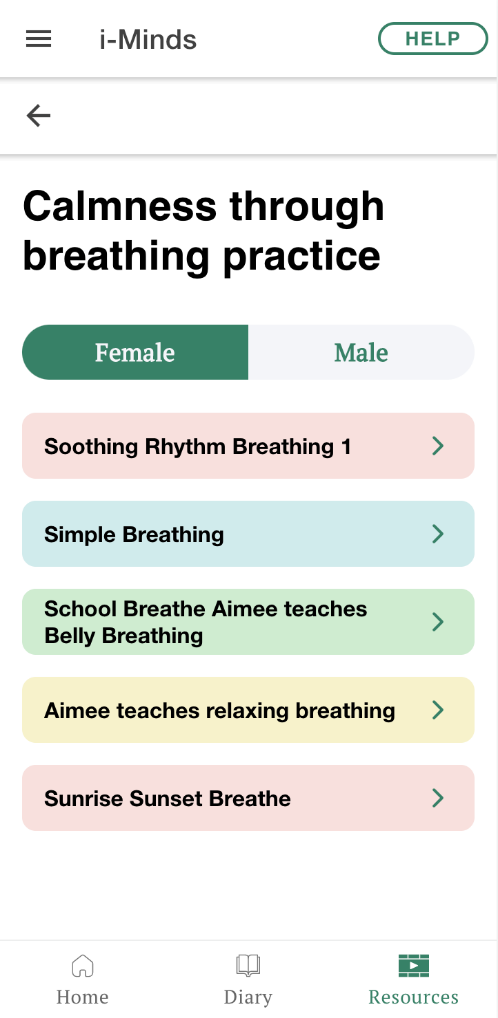


**Supplementary Table 2.** *Content of i-Minds app modules*

| Module | Sub-module | Description |
| --- | --- | --- |
| Module 1: Tech, relationships, and me | | |
|  | **Your rights** | - Explanation of your right to relationships, privacy, and safety. - Link to videos - Quiz checking out key concepts - Explanation of digital rights - Links to videos on digital rights |
|  | **Sexual experiences going wrong?** | - Explanation of how sexual experiences online can go wrong - Link to National Society for the Prevention of Cruelty to Children (NSPCC) video - Effects of these experiences - Link to video about mental health |
|  | **Online relationships** | - Link to video of young people talking about the relationship challenges they face online |
|  | **Being with others can be risky** | - Explanation normalising need for relationships and connection - Link to video about sharing nude images - Exercise inviting users to think about how a person is thinking and feeling during a vignette - Suggested scripts for saying no to sharing images - Link to video from Childnet |
|  | **Me, others and social media** | - Explanation of the impact of comparing self to others on social media - Link to video by Dove - Link to video by Childnet on male perspectives - Exercise encouraging mentalisation - Video about online relationships |
| Module 2: Emotional and mental health | | |
|  | **My feelings and me** | - Information on emotions - Video about feelings - Exercise encouraging mentalisation around feelings / emotions - Video about recognising others feelings |
|  | **Feeling anxious or worried** | - Information about anxiety - Video about anxiety - Exercise introducing feelings thermometer - Exercise encouraging reflection on body sensations, thoughts, and feelings - Video on what to do to help anxiety |
|  | **Self-harm and self-destructive behaviour** | - Information on self-harm and overwhelming feelings - Video explaining self-harm urges - Exercise encouraging mentalisation through a vignette - Video from *The Great Gatsby* showing overwhelming feelings with invited reflection |
|  | **Positive relationships and trusting others** | - Information on healthy and unhealthy relationships - Video link to the film *Inside Out* - Information on patterns of relating - Video illustrating relationship patterns - Exercise encouraging mentalisation through perspective taking - Video showing development of grooming relationships online |
|  | **Developing resilience** | - Normalisation of experiences of stress - Video about effects of stress - Exercise encouraging mentalisation through perspective taking - Video on link between strong feelings and how we act |
| Module 3: Dealing with the past (trauma) | | |
|  | **Triggers** | - Education about triggers - Video explaining triggers - Exercise encouraging mentalisation through perspective taking - Quiz checking out key concepts - Video about how to cope with triggers |
|  | **Understanding and working with guilt and shame** | - Information and normalisation of trauma responses - Video explaining why we might feel guilt and shame - Exercise encouraging mentalisation through perspective taking - Video about neurobiology |
|  | **Avoidance** | - Explanation on how avoidance develops - Video about avoidance - Exercise developing a fear ladder for a vignette - Video about reducing avoidance |

**Supplementary Figure 2.** Total time spent using each app feature


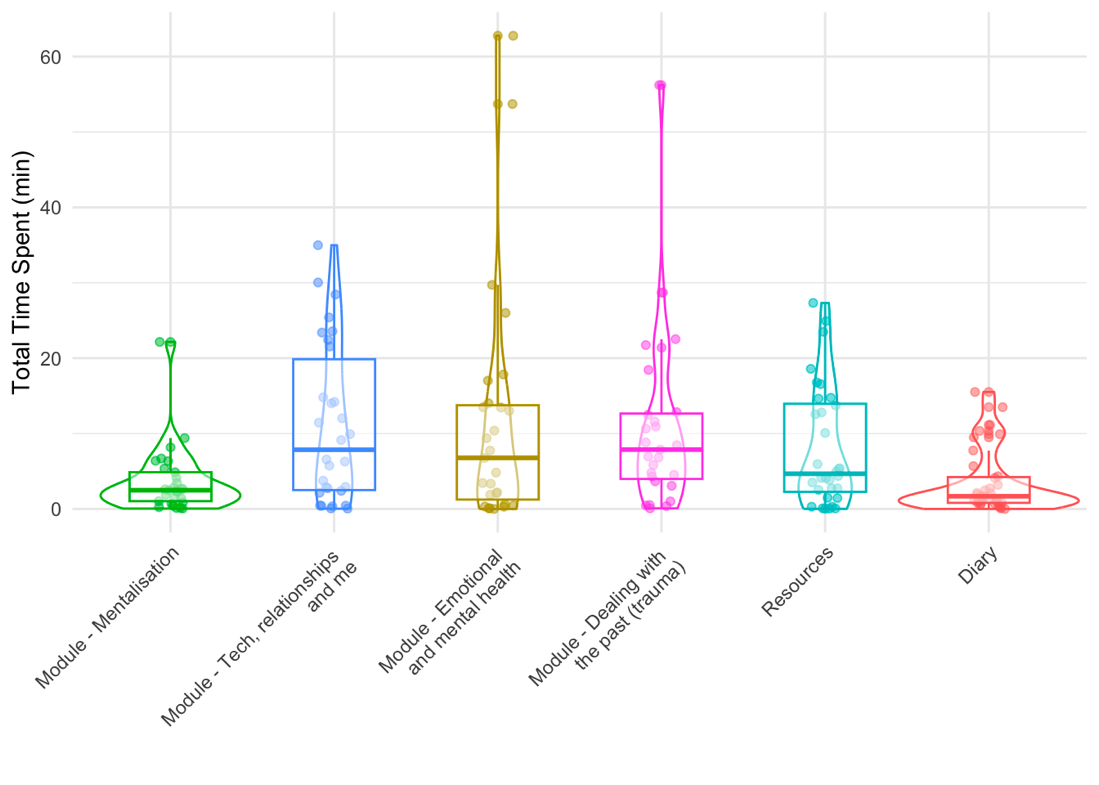


*Caption:* Scatter plot shows the total time spent on specific app contents of each participant (data points were jittered to reduce overlapping); box plot and violin plot show the distribution of the data points.

**Supplementary Figure 3.** Daily total time spent using the app over 6weeks


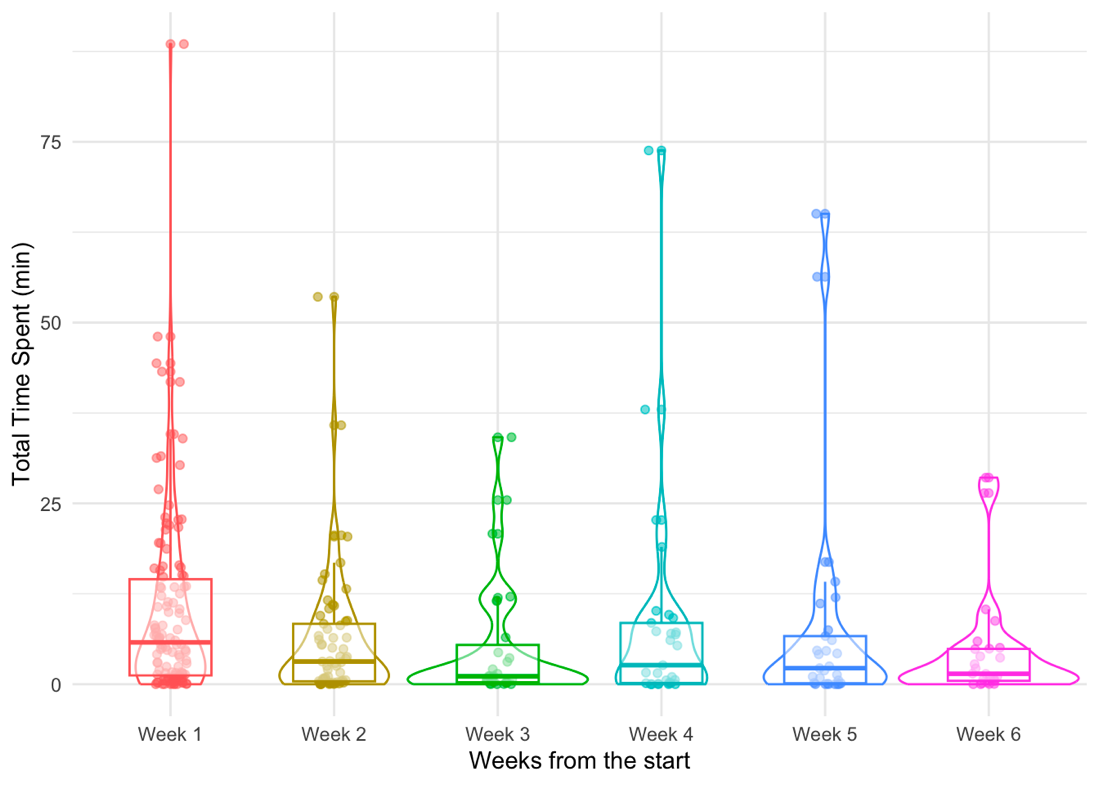


*Caption:* Scatter plot shows the daily total time spent of each participant in each week (data points were jittered to reduce overlapping); box plot and violin plot show the distribution of the data points.
